# Supplementary material for: Quercetin Administration Suppresses the Cytokine Storm in Myeloid and Plasmacytoid Dendritic Cells
Source: Int J Mol Sci. 2021 Aug 3;22(15):8349. doi: 10.3390/ijms22158349 (PMC8348289; doi:10.3390/ijms22158349)
Supplement: Supplementary file 1 [file ijms-22-08349-s001.zip › ijms-1316687-supplementary.pdf]

SUPPLEMENTARY MATERIAL

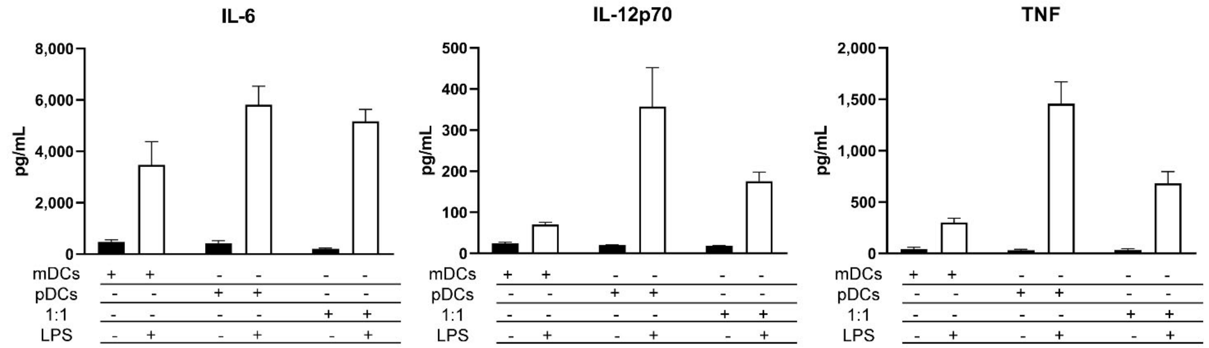

Figure S1. Bar plots expressing the mean ± SEM (n=3) for secreted cytokines of mDCs, pDCs and 1:1 culture in baseline conditions and after stimulation with LPS.

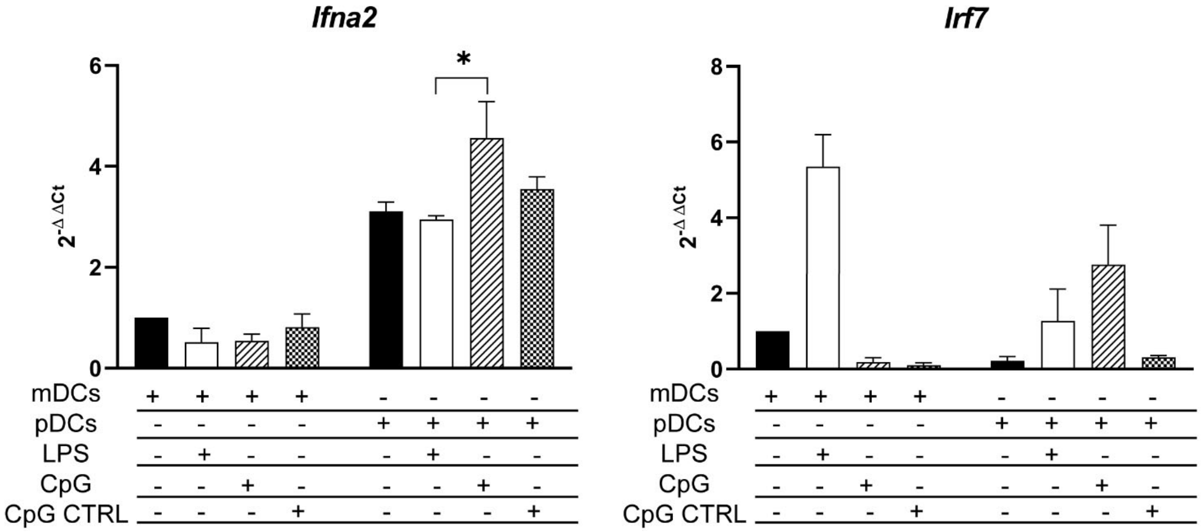

Figure S2. Gene expression profiles of mDCs and pDCs 6h after LPS or CpG or CpG control stimulation. Bar plots representing the mean ± SEM (n=3) relative to control mDCs. *p < 0.05.
